# *Pistacia vera* L. as natural source against antimicrobial and antiviral resistance

**DOI:** 10.3389/fmicb.2024.1396514

**Published:** 2024-07-01

**Authors:** Giuseppina Mandalari, Rosamaria Pennisi, Teresa Gervasi, Maria Teresa Sciortino

**Affiliations:** ^1^Department of Chemical, Biological, Pharmaceutical, and Environmental Science, University of Messina, Messina, Italy; ^2^Department of Biomedical and Dental Science and Morphofunctional Imaging, University of Messina, Messina, Italy

**Keywords:** pistachio, bioactive compounds, antimicrobial effect, antivirals, mechanism of action

## Abstract

Increased global research is focused on the development of novel therapeutics to combat antimicrobial and antiviral resistance. Pistachio nuts represent a good source of protein, fiber, monounsaturated fatty acids, minerals, vitamins, and phytochemicals (carotenoids, phenolic acids, flavonoids and anthocyanins). The phytochemicals found in pistachios are structurally diverse compounds with antimicrobial and antiviral potential, demonstrated as individual compounds, extracts and complexed into nanoparticles. Synergistic effects have also been reported in combination with existing drugs. Here we report an overview of the antimicrobial and antiviral potential of pistachio nuts: studies show that Gram-positive bacterial strains, such as *Staphylococcus aureus*, are the most susceptible amongst bacteria, whereas antiviral effect has been reported against herpes simplex virus 1 (HSV-1). Amongst the known pistachio compounds, zeaxanthin has been shown to affect both HSV-1 attachment penetration of human cells and viral DNA synthesis. These data suggest that pistachio extracts and derivatives could be used for the topical treatment of *S. aureus* skin infections and ocular herpes infections.

## 1 Introduction

Tackling antimicrobial resistance (AMR), considered a threat to global human health, is a key research priority. The term ‘antimicrobial’ includes antibiotic, antiprotozoal, antiviral and antifungal medicines. AMR causes an estimated death of at least 1.27 million people worldwide and was associated with nearly 5 million deaths in 2019, according to a recent report released in The Lancet (Murray et al., 2022). In the U.S., more than 2.8 million antimicrobial-resistant infections occur each year, resulting in the death of more than 35,000 people (CDC, 2019). The estimated national cost in the U.S. to treat infections caused by multidrug-resistant pathogens frequently found in hospital environments can be substantial, leading to more than $4.6 billion annually. Dedicated prevention and infection control efforts can reduce the impact of antimicrobial-resistant infections, lowering deaths by an average of 18% (and by nearly 30% in hospitals in the U.S. in 2019). However, the COVID-19 pandemic has harmed recent AMR prevention and control. Therefore, the United Nations and the World Health Organization are leading initiatives to raise awareness of the problem and deliver solutions to protect our ability to fight infectious diseases globally. Therefore, the discovery of novel therapeutics with antimicrobial and antiviral effects, to be used either alone or in combination with existing drugs, is warranted.

It is well known that plant extracts represent an important source of bioactive compounds, mainly secondary metabolites, which could be used for their antimicrobial and antiviral potential. Tree nuts are known to contain an array of phytochemicals with potential health benefits (Gervasi et al., 2021). However, the phytochemical content of tree nuts can vary considerably, and depends on nut species, genotype, pre- and post-harvest conditions, and storage conditions (Bolling et al., 2011). Additionally, processing approaches such as roasting, may affect tree nut phytochemicals. For example, the total phenol content and the ferric-reducing antioxidant power (FRAP) value both decreased in almond skins after roasting, although the flavonoids concentration was not affected (Bolling et al., 2010a). The choice of solvents used for polyphenols and phytosterols extraction also significantly affects their quantification, and biological activity (Ghırardello et al., 2010). Qasimi et al. (2016) tested the effect of five different solvents (water, 80% methanol, 80% ethanol, acetone and chloroform) on the polyphenols and proanthocyanidin quantification as well as the biological potential [total phenolic (TPC), total flavonoid (TFC, antioxidant capacity using DPPH radical scavenging and ferric reducing antioxidant power (FRAP) activities] of medicinal alophytes: results showed that 80% methanol was the most effective solvent, followed by ethanol and water. Pistachio (*Pistacia vera* L.) nuts represent a good source of protein, fiber, monounsaturated fatty acids, minerals, and vitamins, as well as carotenoids, phenolic acids, flavonoids and anthocyanins (Mandalari et al., 2021). The phytochemical fraction in pistachios is known to contribute to their antimicrobial and antiviral effect and could help overcome AMR (Mandalari et al., 2021).

Here, we present an overview of the antimicrobial and antiviral potential of pistachio nuts. In addition, the evaluation of pistachio polyphenols, alone or in combination with existing drugs, is described in this review in an attempt to provide novel tools to combat AMR.

## 2 Antimicrobial and antiviral properties of pistachio bioactives

Pistachios are known to contain an array of bioactive compounds, which include carotenoids^1^ [beta-carotene, alpha-carotene, lutein and zeaxanthin, chlorophylls (chlorophyll a, chlorophyll b, pheaphytin a)] (Giuffrida et al., 2006; Bellomo and Fallico, 2007), gamma-tocopherol, gamma-tocotrienol, phytosterols^1^ (campsterol, beta-sitosterol, stigmasterol), phenolic acids^1,^^2^ (Neveu et al., 2010), resveratrol (Tokuşoglu et al., 2005; Gentile et al., 2007; Grippi et al., 2008), flavonoids^1,2^ (Tomaino et al., 2010), anthocyanins (cyanidin-3-galactoside, cyanidin-3-glucoside) (Seeram et al., 2008), proanthocyanidins (PAC) and stilbenes^1,^^3^, isoflavones (genistein, genistein-7-*O*-glucoside, dadzein) (Bulló et al., 2015), as well as phytates, sphingolipids, alkylphenols and lignans (Bolling et al., 2010b). The total phenolic content (TPC, mg gallic acid equivalent/100 g, fresh weight) in pistachios has been reported to between 1657 (USDA) and 1,420 (Phenol-Explorer), where the most abundant phenolic compounds were: daidzein, genistein, quercetin, eriodictyol, luteolin, naringenin, and cyanidins (amongst flavonoids); gallic acid and derivatives including gallotannins; alkyl phenols^4^^,^^5^ (anacardic acid derivatives) (Yang et al., 2009).

Current research shows the principal health-related properties of bioactive compounds found in pistachios including their antioxidant and cytoprotective effects, their effect on cell redox homeostasis, as well as their anti-inflammatory and anti-cancer effects, neuroprotective, anti-obesity and anti-diabetic effects (Maestri, 2023). Additionally, a growing body of research has documented antimicrobial and antiviral properties associated with numerous pistachio compounds, as reported in Table 1 (carotenoids, chlorophylls, gamma tocopherol, phytosterols and resveratrol) and Table 2 (flavonoids, isoflavones, anthocyanins and proanthocyanidins). Here, we discuss the antimicrobial and antiviral properties of each of these classes of compounds in more detail.

**Table 1:** Antimicrobial and antiviral effects of pistachio carotenoids, chlorophylls, gamma tocopherol, phytosterols and resveratrol.

Compound	Antimicrobial effect	Active antimicrobial concentration	Antiviral effect	Active antiviral concentration	Reference
Beta-carotene	*Klebsiella pneumonia, Escherichia coli, Staphylococcus aureus* *Salmonella enteritidis*	100 mg/mL 3.8 log units			Abdulhadi et al. (2020) Hayashi et al. (2012)
Lutein	*Enterococcus faecium, Staphylococcus saprophyticus, Staphylococcus aureus, Escherichia coli, Pseudomonas aeruginosa, Klebsiella pneumoniae*	8.0–256.0 μg/mL	Hepatitis B	40 μg/mL	Mitra et al. (2021) Pang et al. (2010)
Zeaxanthin			Herpes simplex virus 1	10 μg/mL	Pennisi et al. (2023)
Chlorophylls	Gram-positive and Gram-negative bacteria	Photosensitizing effect	SARS-CoV-2	> 65 nM	Bertoloni et al. (1992), Jimenez-Aleman et al. (2021)
Gamma tocopherol	*Escherichia coli, Pseudomonas aeruginosa, Bacillus subtilis, Staphylococcus aureus, Chromobacterium violaceum Erwinia carotovora*	0.25–2.0% (v/v)			Ulusoy et al., 2009
Campesterol	*Staphylococcus aureus*, *Streptococcus mutans*, *Escherichia coli*, *Pseudomonas aeruginosa*, *Klebsiella pneumoniae*	0.4–1.3 mM	Herpes simplex virus 2	300 μg/mL	Freitas da Silva et al. (2023) Alqurashi et al. (2022) Jamhour et al. (2022)
			SARS-CoV-2	-	
Beta-sitosterol	*Vibrio* species *Staphylococcus aureus*	10 μg/mL 10 μg/mL	Herpes simplex virus 2	2.7 μg/mL	Ravi et al. (2020) Toujani et al. (2018), Alqurashi et al. (2022)
Stigmasterol	*S. typhi* *K.pneumoniae* *Candida albicans* *Candida krusei*	100 μg/mL 12.5–50.0 μg/mL	Herpes simplex virus 2	5 μM and 10 μM	Petrera et al., 2014 Yusuf et al. (2018) Jamhour et al. (2022) Djouonzo et al. (2023)
			SARS-CoV-2	36.45 μg/mL	
Resveratrol	Gram-positive and Gram-negative bacteria, Fungi	25.0–128.0 μg/mL 10.0–50.0 μg/mL	Influenza virus	10–20 μg/mL	Abedini et al. (2021)
			Rhinovirus	10–50 μM	
			Respiratory syncytial virus	30 mg/kg body weight	
			Coronavirus	62.5 μM	

**Table 2:** Antimicrobial and antiviral effects of pistachio flavonoids, isoflavones, anthocyanins and proanthocyanidins.

Compound	Antimicrobial effect	Active antimicrobial concentration	Antiviral effect	Active antiviral concentration	Reference
Catechin	*Escherichia coli, Salmonella* sp., *Staphylococcus aureus*	6–50 mg/mL 0.15 mg/mL	Herpes simplex virus 1	0.4 mg/mL	Ma et al. (2019) Musarra-Pizzo et al. (2019)
Epicatechin	*Staphylococcus aureus*, *Helicobacter pylori*	0.078–0.15 mg/mL 128–1,024 μg/mL	Herpes simplex virus 1	0.4 mg/mL	Taylor et al. (2005) Reygaert (2014) Musarra-Pizzo et al. (2019) Bisignano et al. (2013b)
Quercetin	Gram-positive and Gram-negative bacteria Fungi	0.002–8 mg/mL 16–64 μM	Flaviviridae	50 μM	Rojas et al. (2016) Anand David et al. (2016) Nguyen and Bhattacharya, (2022) Di Petrillo et al. (2022) Fanunza et al. (2020)
			Orthomyxoviridae	1.2 μM	
			Herpesviridae	145 μM	
			Coronaviridae	200 μM	
			Retroviridae	11.0 μM	
			Enterovirus 71 (EV71)	39.63 μg/mL	
			Coxsackievirus A16 (CVA16)	59.53 μg/mL	
			Filoviridae	7.4 μM	
Eriodictyol	*Escherichia coli, Salmonella enterica, Pseudomonas putida, Bacillus subtilis, Listeria innocua, Lactococcus lactis, Staphylococcus aureus, Saccharomyces cerevisiae*	250–800 μg/mL	SARS-CoV-2	10 μM	Mandalari et al. (2007), Kaul et al. (2021)
Naringenin	*Staphylococcus aureus, Listeria monocytogenes, Salmonella enterica*	250–500 μg/mL	SARS-CoV-2	200 μM	Mandalari et al. (2010), Kaul et al. (2021)
Kaempferol	Gram-positive and Gram-negative bacteria Fungi	0.5-625 μg/mL 25-500 μg/mL	SARS-CoV-2	200 μM	Periferakis et al. (2022), Kaul et al. (2021)
Apigenin	Gram-positive and Gram-negative bacteria Fungi		Herpes simplex virus 1	5 μg/mL	Wang et al. (2019) Lee et al. (2023) Kaul et al. (2021)
			Enterovirus 71	11.0 μM	
		2–400 μg/mL	Hepatitis C virus	0.1 μM- 5 μM	
		8–68 μg/mL	Dengue virus	40 μM	
			SARS-CoV-2	200 μM	
			Influenza virus	1.438 μg/mL- 15.3 μg/mL	
Luteolin	*Staphylococcus aureus* *Listeria monocytogenes*		Coronavirus	10.6 μM	Yi et al. (2004) Xu et al. (2014) Qian et al. (2020) Banerjee et al. (2022) Lu et al. (2023) Wang et al. (2023) Wang et al. (2020)
		16–32 μg/mL	Influenza virus	73 ± 3 nM	
		32–64 μg/mL	Enterovirus 71	10.31 μM	
			Rotavirus	2.79–4.36 mM	
			Herpes virus	25 μM	
			Respiratory syncytial virus	2.075–49.94 μM	
Genistein	*Staphylococcus aureus, Bacillus cereus*	62.5–1,000 μM	Herpes B virus	33 μM	Hong et al. (2006) LeCher et al. (2019) Vela et al. (2010)
			Hemorrhagic fever virus	50 μM - 100 μM	
Daidzein	*Staphylococcus aureus* (ATCC and clinical isolates)	2,048–4,096 μg/mL	Hepatitis C virus	50 μM	Lalouckova et al. (2021) He et al. (2021)
Anthocyanins	*Escherichia coli, Salmonella* sp. *Staphylococcus aureus, Listeria monocytogenes*		Herpes simplex virus 1	> 20 μg/mL	Choudhary and Pan (2020) Ma et al. (2019) El Majdoub et al. (2021), Mohammadi Pour et al. (2019) Hayashi et al. (2003)
		10–400 mg/mL	Coxsackievirus B1	–	
			Influenza A virus	48 mug/ml	
			Influenza B virus	54 mug/ml	
			Avian influenza virus	–	

### 2.1 Carotenoids

Carotenoids are tetraterpene pigments which exhibit orange, yellow, red and purple colours. Amongst the carotenoids present in pistachio nuts, β-carotene at a concentration of 100 mg/mL showed the best antimicrobial activity against the bacterial species *Klebsiella pneumoniae* (inhibition zone diameter of 40 mm), followed by *Escherichia coli* and *Staphylococcus aureus* (inhibition zone diameter of 36 and 31 mm, respectively, Abdulhadi et al. (2020)). However, no effect was shown against *Pseudomonas aeruginosa*. In addition, β -carotene has been found to induce an increase from 1.4 to 3.8 log units in the bactericidal activity of a bovine lactoperoxidase system, evaluated using *Salmonella enteritidis* (Hayashi et al., 2012).

According to Mitra et al. (2021), the carotenoid pigment lutein was able to inhibit both the growth and the proliferation of several Gram-positive and Gram-negative bacteria, such as *Enterococcus faecium*, *S. saprophyticus*, *S. aureus*, *E. coli*, *P. aeruginosa*, *K. pneumoniae*, at concentrations varying from 8 to 256 μg/mL. The *in vitro* antiviral activity of lutein against the hepatitis B virus (HBV) has been reported by Pang et al. (2010). The antiviral functions of lutein have also been investigated in stable HBV-producing human hepatoblastoma HepG2 2.2.15 cells, where it efficiently suppressed the dose-dependent secretion of HBsAg and inhibited extracellular HBV DNA. We have recently reported that zeaxanthin, a dietary carotenoid that accumulates in the retina as a macular pigment, exhibits strong antiviral activity against herpes simplex virus 1 (HSV-1, CC50: 16.1 μM, EC50 4.08 μM, SI 3.96), affecting viral attachment and penetration as well as viral DNA synthesis (Pennisi et al., 2023). An overview of the use of carotenoids as therapeutic strategies against emerging viral diseases, such as COVID-19, has recently been published (Khalil et al., 2021). The inhibitory activity of two marine carotenoids blocking the entry of SARS-CoV-2 has also been reported (Yim et al., 2021).

### 2.2 Tocopherols

Tocopherols are a class of organic fat-soluble phenolic compounds, many of which have vitamin E activity. The antibacterial potential of gamma-tocopherol has been demonstrated in flower extracts of Damask rose (Rosa damascena Mill), which contained beta-carotene, alpha-tocopherol and phenolic compounds as well as gamma-tocopherol: results demonstrated a strong antibacterial activity against *E. coli* (ATCC 25922), *P. aeruginosa* (ATCC 27853), *Bacillus subtilis* (ATCC 6633), *S. aureus* (ATCC 6538), *Chromobacterium violaceum* (ATCC 12472) and *Erwinia carotovora* (ATCC 39048) strains (Ulusoy et al., 2009).

### 2.3 Chlorophylls

The susceptibility of Gram-positive and Gram-negative bacteria to photodynamic therapy using natural chlorophylls, the green photosynthetic pigments found in plants, has been widely investigated (Bertoloni et al., 1992; Merchat et al., 1996; Minnock et al., 1996). The use of derivatives of natural chlorophylls as agents for antimicrobial photodynamic therapy has recently been reviewed also in relation to their effect against bacterial biofilm, which is known to be highly resistant to antibiotic treatment (Suvorov et al., 2021). A chlorophyll derivative pheophorbide A (PheoA), a porphyrin compound similar to animal Protoporphyrin IX, has shown an antiviral activity against SARS-CoV-2, preventing infection of cultured monkey and human cells, without noticeable cytotoxicity (Jimenez-Aleman et al., 2021).

### 2.4 Phytosterols

Phytosterols are plant-based compounds similar to cholesterol. Using the microdilution method, the phytosterol campsterol displayed a weak antibacterial effect *in vitro* against *S. aureus* (ATCC 6538), *Streptococcus mutans* (ATCC 0046), *E. coli* (ATCC 10536), *P. aeruginosa* (ATCC 15442), and *K. pneumoniae* (ATCC 10031), with minimum inhibitory concentration (MIC) values of 1.280 mM (Freitas da Silva et al., 2023). Through computational analyses, it was proposed that β-sitosterol exhibited antibacterial activity against several bacterial *Vibrio* species and could be used in aquaculture, both as a nutritional supplement and also as a disease control agent to prevent and control fish diseases caused by bacterial infection (Ravi et al., 2020). Furthermore, β-sitosterol isolated from the leaves of the South American firespike plant (*Odontonema strictum*) has been found to be active against *S. aureus*, showing both a bacteriostatic and a bactericidal effect (Pierre Luhata and Usuki, 2021). Yusuf et al. (2018) have demonstrated the antibacterial and the antifungal activity of stigmasterol isolated from the stem bark of the African tree species Neocarya macrophylla (Yusuf et al., 2018).

Seed oil from the prickly pear (*Opuntia ficus-indica*) containing phytosterols, primarily campesterol, followed by γ- & β -sitosterol, and stigmasterol, has been shown to exhibit an antiviral effect (22.67 ± 2.79%) at 300 μg/mL of oil against herpes simplex type 2 (HSV-2) virus (Alqurashi et al., 2022). After rutin, stigmasterol and campesterol were shown to be the most prominent inhibitors for SARS-CoV-2 proteins using an *in-silico* approach (Jamhour et al., 2022).

### 2.5 Resveratrol

Resveratrol is a natural phenolic compound with antioxidant-like properties. A comprehensive study on the antimicrobial and antiviral properties of resveratrol as an alternative therapy has recently been published by Abedini et al. (2021) (Abedini et al., 2021). Resveratrol has been shown to inhibit the growth of numerous bacteria detrimental to human health, including *B. cereus* species (at a concentration of 50 μg/mL) (Paulo et al., 2010), *Mycobacterium smegmatis* (64 μg/mL) (Lechner et al., 2008), *Helicobacter pylori* (25–50 μg/mL) (Makobongo et al., 2014), *Vibrio cholerae* (60 μg/mL) (Augustine et al., 2014), *Neisseria gonorrhoeae* (75 μg/mL) (Vestergaard and Ingmer, 2019), *Campylobacter coli* (50 μg/mL) (Duarte et al., 2015), and *Arcobacter cryaerophilus* (50 μg/mL) (Ferreira et al., 2014). An antifungal activity of resveratrol has been reported *in vitro* against the yeast *Candida albicans* at a concentration of 20 μg/mL (Lee and Lee, 2015). Furthermore, resveratrol has been shown to have inhibitory activity against viral replication and viral-induced inflammation by several respiratory viruses, including influenza virus (Pourghanbari et al., 2016), respiratory syncytial virus (Liu et al., 2014), coronavirus (SARS-CoV and MERS-CoV) (Filardo et al., 2020), and rhinovirus (Mastromarino et al., 2015).

### 2.6 Flavonoids

Flavonoids are polyphenolic secondary metabolites found in plants. Extensive scientific literature is available on the antimicrobial and antiviral properties of catechins and epicatechins (Taylor et al., 2005; Reygaert, 2014). For example, epicatechin-3-gallate, epigallocatechin, and epigallocatechin-3-gallate have been shown to have antimicrobial effects against a variety of bacteria, including *S. aureus*, methicillin-resistant *S. aureus* (MRSA) and *E. coli* (Reygaert, 2014). Taylor et al. (2005) demonstrated that low concentrations of epicatechin gallate can sensitize MRSA clinical isolates to levels of oxacillin which can be readily achieved in clinical practice.

We have previously demonstrated the antibacterial effect of catechin and epicatechin against *S. aureus* ATCC 6538P (MIC values of 0.078–0.15 and 0.15 mg/mL, respectively) and *H. pylori* (both ATCC strains and clinical isolates, Bisignano et al. (2013b)) and the antiviral activity (decrease in the viral titer ** *p* < 0.01, and viral DNA accumulation * *p* < 0.05) of a polyphenols mix containing catechin, naringenin-7-O-glucoside, kaempferol-3-O-glucoside, epicatechin, isorhamnetin-3-O-rutinoside, and isorhamnetin-3-O-glucoside against HSV-1 (Musarra-Pizzo et al., 2019). Through Western blot, real-time polymerase chain reaction (PCR) and viral binding assay, we also demonstrated that polyphenols were able to block the production of infectious HSV-1 particles and inhibited HSV-1 adsorption to Vero cells (Bisignano et al., 2017).

The antimicrobial and antiviral activity of quercetin, and the possible mechanism of action, have recently been reviewed (Di Petrillo et al., 2022; Nguyen and Bhattacharya, 2022). Strong growth inhibition of different Gram-positive and Gram-negative bacteria has been reported, particularly affecting gastrointestinal, respiratory, urinary, and dermal system (Anand David et al., 2016) as well as the yeast *C. albicans* (Singh et al., 2015; Gao et al., 2016) the fungi *Aspergillus fumigatus* (Yin et al., 2021) and *Aspergillus niger* (Abd-Allah et al., 2015) and several viruses, such as the human immunodeficiency virus (HIV)-1 strain (Kim et al., 1998), the herpes simplex and the respiratory syncytial virus (Cushnie and Lamb, 2005), the polio-virus type 1 (Kaul et al., 1985) and the influenza virus (Liu et al., 2016). The mechanism of quercetin antimicrobial action includes cell membrane damage, change of membrane permeability, inhibition of synthesis of nucleic acids and proteins, reduction of expression of virulence factors, mitochondrial dysfunction, and prevention of biofilm formation.

We have previously reported the antibacterial potential of eriodictyol and naringenin: eriodictyol was more effective (MICs in the range of 250 and 800 μg/mL) compared with other tested flavonoids, such as naringenin, against a range of Gram-positive and Gram-negative bacteria (*E. coli, Salm. enterica, P. putida, B. subtilis, L. innocua, Lactococcus lactis, S. aureus*) and the yeast *Saccharomyces cerevisiae* (Mandalari et al., 2007). On the other hand, naringenin was active against *S. aureus* ATCC 6538P, *L. monocytogenes* ATCC 7466 and *Salm. enterica* ser. Typhimurium ATCC 14028 (MIC values of 250, 500 and 250 μg/mL), respectively (Mandalari et al., 2010). Periferakis et al. (2022) reviewed the antibacterial and antifungal properties of kaempferol. Pure kaempferol, kaempferol extracts and nanoparticles loaded with kaempferol have shown activity against Gram-negative bacteria, including *Acinetobacter baumannii* (Özçelik et al., 2006; Rofeal et al., 2021), *Enterobacter cloacae* (Christopoulou et al., 2008), *Enterobacter aerogenes* (Karimi et al., 2011), *E. coli* (Özçelik et al., 2006; Kannanoor et al., 2021), *K. pneumoniae* (Habbu et al., 2009), *P. aeruginosa* (Attallah et al., 2022), *Vibrio cholerae* (Martini et al., 2004), as well as Gram-positive bacteria, including *S. aureus* (Özçelik et al., 2006), *Streptococcus pyogenes* (Özçelik et al., 2006), *Bacillus* sp. (Youssef et al., 2021), and *Mycobacterium* sp. (Nguyen et al., 2021). Moreover, both isolated kaempferol-3-O-[3-O-acetyl-6-O-(E)-p-coumaroyl]-b-d-glucopyranoside and kaempferol 3-O-b-D-kaempferol 3-O-b-D-glucopyranoside, were found to be active *in vitro* against *C. albicans*, *Candida glabrata* and *Candida tropicalis* (Christopoulou et al., 2008).

Eriodictyol, naringenin and kaempferol have been identified as promising antiviral compounds against SARS-CoV-2, both alone and together existing antiviral drugs, targeting specifically the promising 3C-like protease (3CLpro) (Kaul et al., 2021).

A wide range of *in vitro* antibacterial activity has been reported for apigenin by Wang et al. (2019): apigenin was active against *Acinetobacter baumannii* (with MIC values between 2 and 64 μg/mL), *Bacillus subtilis* (MIC values between 8 and 16 μg/mL), *Enterococcus faecalis* ATCC 29212 (MIC = 8 μg/mL), *E. coli* ATCC 35818 (MIC = 4 μg/mL), *K. pneumoniae* RSKK 574 (MIC = 8 μg/mL) and ESβL+ clinical isolate (MIC = 128 μg/mL), *Proteus mirabilis* ATCC 7002, (MIC = 4 μg/mL), *S. aureus* ATCC 25923 (MIC = 16 μg/mL), and *C. albicans* ATCC 10231 (MIC = 8 μg/mL) (Özçelik et al., 2011). The therapeutic potential of apigenin against viral infection has recently been reviewed by Lee et al. (2023). Apigenin exerts virucidal activity against HSV-1 interfering with viral absorption and inhibiting the post-entry step of the viral replication (Lyu et al., 2005; Yucharoen, 2011; Visintini Jaime et al., 2013; Fahmy et al., 2020; Rittà et al., 2020), against Enterovirus 71 by inhibiting the interaction between internal ribosome entry site of EV71 and hnRNP A1 and A2 (Shih et al., 2011; Lv et al., 2014; Zhang et al., 2014; Ji et al., 2015; Dai et al., 2019), against hepatitis C virus by binding to NS5B and inhibiting RdRp activity, decreasing miR122 expression levels and suppressing the phosphorylation of TRBP (Manvar et al., 2012; Ohno et al., 2013; Pisonero-Vaquero et al., 2014; Shibata et al., 2014), against Dengue virus by restoring STAT2 Tyr 689 phosphorylation and activation, colocalization with a DENV protein in the early phase of infection (Mazzon et al., 2009; Jasso-Miranda et al., 2019; Acchioni et al., 2023), against SARS-CoV by reducing the production of proinflammatory cytokine in response to viral infection, as well as interacting with viral protein (Mpro) and host factor (ACE-2 receptor, TMPRSS2) (Ryu et al., 2010; Jo et al., 2020; Zhang et al., 2020; Alzaabi et al., 2022; Chaves et al., 2022; Farhat et al., 2022) as well as against influenza virus - by neuroaminidase inhibition, viral attachment and entry inhibition, inhibition of viral mRNA expression, inhibition of influenza A virus RdRP activity, reduction of viral particle production, and nucleoprotein reduction (Liu et al., 2008; Kai et al., 2014; Xu et al., 2020a, 2020b; Joo et al., 2022; Morimoto et al., 2023).

Amongst the flavanones, luteolin has also shown antibacterial potential against *S. aureus* and *L. monocytogenes in vitro*, by impairment effect on the cell membrane and restraining biofilm formation of both strains (Qian et al., 2020). A literature review on the antiviral mechanism of luteolin has recently been published by Lu et al. (2023): luteolin effectively inhibited coronavirus replication (Alzaabi et al., 2022; Chen et al., 2022), influenza virus (Lee et al., 2016), enterovirus (Chen et al., 2008; Cao et al., 2016), rotavirus (Knipping et al., 2012), herpes virus (Lu et al., 2023), and respiratory syncytial virus (Wang et al., 2020). In particular, it prevented viral infection by improving the host’s nonspecific immunity and antioxidation capacity, thus inhibiting several pathways related to viral infection, including MAPK, PI3K-AKT, TLR4/8, NF-κB, and Nrf-2/hemeoxygenase-1. Furthermore, luteolin was able to regulate the expression of specific receptors and factors, interfering with viral replication and thus promoting the repair of damaged cells induced by proinflammatory factors (Lu et al., 2023).

Hong et al. (2006) demonstrated *in vitro* the antibacterial potential of the isoflavone genistein against the Gram-positive *S. aureus* and *Bacillus anthracis* strains (Hong et al., 2006). Furthermore, an investigation on the mechanism of action of genistein indicated altered cell morphology (formation of filamentous cells) on bacterial cells, together with an inhibition of DNA and RNA synthesis as shortly as 15 min after addition to a bacterial culture. Protein synthesis inhibition was also detected (Ulanowska et al., 2006). Genistein has also been shown to possess antiviral activity against the herpes B virus, acting synergistically with existing antiviral drugs (LeCher et al., 2019). An effect of genistein as a general kinase inhibitor against an arenaviral haemorrhagic fever surrogate virus has also been demonstrated (Vela et al., 2010).

The antibacterial and antifungal effect of the isoflavone daidzein has been reported against *S. aureus* (both ATCC and clinical strains) by Lalouckova et al. (2021). Dietary daidzein was able to inhibit hepatitis C virus replication by decreasing microRNA-122 levels (He et al., 2021).

### 2.7 Anthocyanins

Anthocyanins are a group of red and blue pigments found in plants and along with catechins, form subgroups within the flavonoids. The antimicrobial activity of anthocyanins and catechins against the foodborne pathogens *E. coli* and *Salmonella* sp. has been reported, with MIC values between 10–400 mg/mL (Ma et al., 2019). Mechanistically, anthocyanins can act as an antibacterial by destroying the cell wall of foodborne pathogens: anthocyanins extracted from the Assegai tree (*Curtisia dentata*) were able to destroy the *E. coli* cell wall (Doughari et al., 2012), whereas in another study, anthocyanins extracted from lowbush wild blueberries were able to destroy the cell membranes of *E. coli* O157: H7, with consequent cytoplasmic leakage (Lacombe et al., 2013). Although anthocyanins are active against different bacterial strains, Gram-positive bacteria are usually more susceptible than Gram-negative bacteria (Cisowska et al., 2011). We have demonstrated the effect of an anthocyanin extract from the flowering plant *Roselle* (*Hibiscus sabdariffa* L.) containing cyanidin-3-O-sambubioside and delphinidin-3-O-sambubioside against *S. aureus* ATCC 6538 and against a food isolate of *L. monocytogenes*. The effect was bacteriostatic against *S. aureus* (MIC = 2.5 mg/mL) and bactericidal against *L. monocytogenes* (Minimal Bactericidal Concentration, MBC = 2.5 mg/mL) (El Majdoub et al., 2021).

The antiviral potential of anthocyanidins has been recently reviewed by Mohammadi Pour et al. (2019): a total anthocyanins extract from strawberry (*Fragaria x ananassa*) was active against HSV-1 (Simões et al., 2012), whereas an anthocyanins extract from wild strawberry (*Fragaria vesca*), raspberry (*Rubus idaeus*), blueberry (*Vaccinium myrtillis*) and lingonberry (*Vaccinium vitis-idaea*) was active against coxsackievirus B1 (CV-B1) and influenza A virus (Nikolaeva-Glomb et al., 2014). Specifically, cyanidin-3-galactoside, contained in pistachios, was active against the influenza A, influenza B and Avian influenza viruses by Increasing the NK cell activity and enhancing the immune system responses (Niemenak et al., 2006).

These reports illustrate the extensive antimicrobial and antiviral effects of pistachio phytochemicals, isolated or within plant extracts, alone and combined with existing drugs. In terms of antibacterial activity, Gram-positive strains were usually more susceptible than Gram-negative bacteria, with a mostly bacteriostatic rather than bactericidal effect. Amongst the Gram-positive bacterial strains tested, ATCC and clinical isolates of *S. aureus* were more susceptible to the effect of bioactive compounds. *S. aureus* and MRSA are known to be responsible of various infections, including biofilm-associated diseases, ranging from skin, prostheses, catheters and other biomaterials infections to more serious systemic diseases, such as endocarditis, pneumonia, and osteomyelitis. Given the increased incidence of antibiotic resistant *S. aureus* infections, especially within hospital settings, the discovery of natural compounds effective against *S. aureus* produced biofilm is a promising area for further clinical research (Mandalari et al., 2023).

## 3 Antimicrobial effect of pistachios and mechanism of action

Table 3 reports the antimicrobial effect of pistachio extracts and essential oil. We have previously demonstrated that polyphenol-rich extracts of natural raw shelled and roasted salted pistachios were active *in vitro* against a range of Gram-positive bacteria, with a bactericidal effect against ATCC strains and food isolates of *L. monocytogenes*, *S. aureus* and MRSA (Bisignano et al., 2013a). Furthermore, we have phenotypically characterized clinical isolates of *Staphylococcus* spp. and tested these for their sensitivity against natural raw and roasted salted pistachios: both extracts were active against clinical isolates of *Staphylococcus* sp., as well as the *S. aureus* ATCC 6538P (La Camera et al., 2018). We have also demonstrated that polyphenol-rich extracts of natural raw shelled and roasted salted pistachios were effective against *L. monocytogenes* food isolate strains (MIC values between 0.25 and 2.0 mg/mL) and against *L. monocytogenes* ATCC 13932 (Gervasi et al., 2022). Furthermore, the oil fractions from natural and roasted pistachios were effective against *L. monocytogenes* ATCC 13932 and *Enterococcus faecium* DSZM 17050. Other authors have confirmed the effect of a methanolic extract of pistachios against staphylococcal infections (Gutiérrez-Morales et al., 2017). *Pistacia vera* L. oleoresin has been tested in combination with levofloxacin, demonstrating a protective effect against *H. pylori* infection in an *in vivo* model of *Galleria mellonella* (62 and 63% survival using oleoresin and levofloxacin, respectively) (Di Lodovico et al., 2019). *Pistacia vera* L. oleoresin was also effective against oral streptococci, such *S. mutans*, with a demonstrated anti-biofilm activity (Magi et al., 2018).

**Table 3:** Antimicrobial effect of pistachio extracts and essential oil.

Pistachio	Strain	Active concentration	Reference
Natural raw and roasted salted polyphenols-rich extracts of *Pistacia vera* L.	*L. monocytogenes*, *S. aureus* and MRSA	15.6–125.0 μg/mL	Bisignano et al. (2013a)
Natural raw and roasted salted polyphenols-rich extracts of *Pistacia vera* L.	*Staph.* spp.	31.2–2000.0 μg/mL	La Camera et al. (2018)
Natural raw and roasted salted polyphenols-rich extracts of *Pistacia vera* L.	*L. monocytogenes* ATCC and food isolates	0.25–2.0 mg/mL	Gervasi et al. (2022)
Methanolic extract of *Pistacia vera* L.	*Staph.* spp.	68.6 ± 0.3% relative percentage inhibition	Gutiérrez-Morales et al. (2017)
*Pistacia vera* L. oleoresin	*H. pylori* infection in an *in vivo* model of *Galleria mellonella*	62% survival rate	Di Lodovico et al. (2019)
*Pistacia vera* L. oleoresin	*Streptococcus* spp.	1024.0–2048.0 μg/mL	Magi et al. (2018)
Essential oil from *Pistacia vera* L. hulls	*S. aureus* and *E. coli*	7.1 mg/ mL	Smeriglio et al. (2017)
Essential oil from *Pistacia vera* L. hulls	*Candida* spp.	2.5–5.0 mg/ mL	D’Arrigo et al. (2019)
Essential oil from *Pistacia vera* L. hulls	*S. aureus, B. subtilis, A. flavus*	60.0–500.0 μg/mL	Shahdadi et al. (2023)
			
Pistachio hull ethanolic (PVE) and aqueous (PVD) extracts	*E. faecalis*, *S. aureus*, *S. uberis*, *B. cereus* and *B. subtilis*	0.8–49.0 (PVE) and 9.6–82.5 (PVD) mg/mL	Seker and Akbas (2023)
			
Pistachio hull extract	*E. coli, B. cereus, S. aureus, P. aeruginosa, A. niger, C. albicans*	0–22 mm growth diameter	Bakhshi et al. (2021)

The essential oil from *Pistacia vera L*. hulls was bactericidal against a range of *S. aureus* strains and *E. coli* at a concentration of 7.11 mg/mL (Smeriglio et al., 2017). A fungicidal effect of pistachio essential oil was demonstrated against standard and clinical strains of *Candida* sp. at concentrations between 2.50 and 5.0 mg/mL, D-limonene and 3-Carene being the most active components (D’Arrigo et al., 2019). The inhibitory activity of pistachio hull essential oil has also recently been demonstrated against *S. aureus*, *B. subtilis* and *Aspergillus flavus* (Shahdadi et al., 2023).

Recently, the phytochemical contents, the antioxidant and antimicrobial activities of pistachio hull ethanolic (PVE) and aqueous (PVD) extracts obtained by microwave-assisted extraction (MAE) were investigated by Seker and Akbas (2023): both extracts showed antimicrobial potential against *E. faecalis*, *S. aureus*, *Streptococcus uberis*, *B. cereus* and *B. subtilis*, with MICs values between 0.8–49.0 and 9.6–82.5 mg/mL and MBC values ranging from 1.3–99.1 and 15.5–150.0 mg/mL for PVE and PVD, respectively (Seker and Akbas, 2023). The antibacterial properties of pistachio hull extracts have also been reviewed by Arjeh et al. (2020).

A pistachio hull extract has been used as a reducing and stabilizing agent with antibacterial and antifungal effects within copper nanoparticles (Bakhshi et al., 2021).

Overall, these studies demonstrate that pistachios extract and essential oil, alone or in association with existing drugs, could be considered good candidates for the development of novel drug formulations. Their activity against Gram-positive bacterial strains, including *S. aureus*, could be exploited to identify novel therapeutics with topical use (i.e., to treat skin infections).

**Figure 1:**
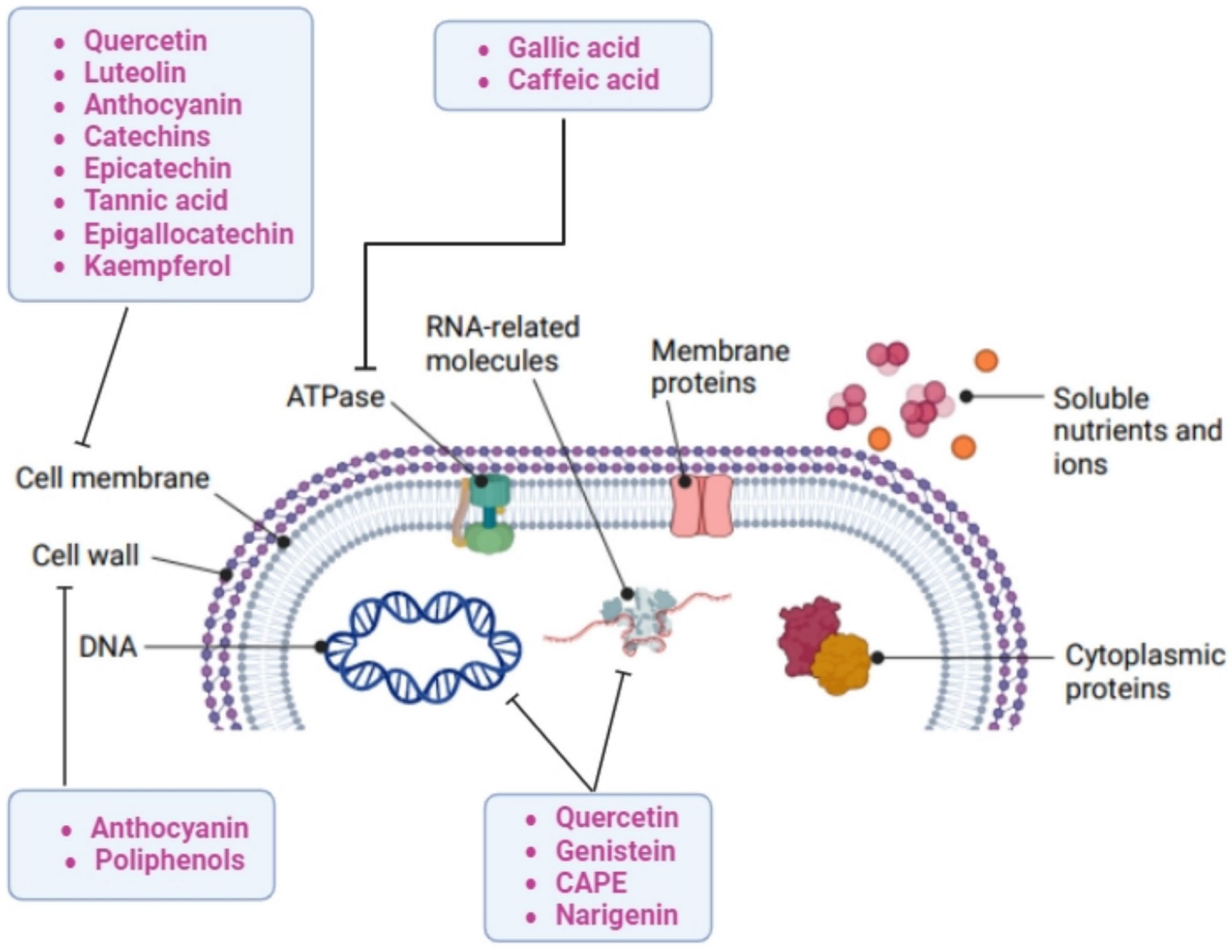
Proposed bacterial molecular targets of bioactives in pistachios.

Although further studies are warranted to evaluate the mechanisms of action involved in the observed effect exerted by pistachio extracts, Figure 1 reports some proposed molecular bacterial targets, as reviewed by Álvarez-Martínez et al. (Álvarez-Martínez et al., 2020). Polyphenols can target the bacterial cell wall, causing morphological damage to the cells, or destroying the structural integrity of the cell wall and intracellular matrix (Din et al., 2013; Pojer et al., 2013).

The different cell wall structure between Gram-positive and Gram-negative bacteria could explain the higher susceptibility of Gram-positive strains to phytochemical antimicrobial activity, given that the outer membrane of Gram-negative bacteria acts as a permeability barrier, therefore reducing the uptake of the phenolic compounds (Naz et al., 2007). Polyphenols can cause leakage by increased permeability of the bacterial membrane and the cell wall (Lambert et al., 2001; Wang et al., 2017). Specifically, catechins can cause destruction of the Gram-positive bacterial membranes through interaction with lipids, which determine phase separations (Reygaert, 2014). Furthermore, epicatechin, tannic acid, epigallocatechin gallate, quercetin and kaempferol demonstrated significant β -lactamase inhibitory activity, also in synergy with antibiotics such as ciprofloxacin and rifampicin (Lin et al., 2008; Bernal et al., 2010; Mandal et al., 2017). Other possible targets could be represented by cell surface adhesion proteins, membrane-bound enzymes and cell wall polypeptides (Naz et al., 2007). A bioactive fraction from the tree species *Duabanga grandiflora* fruit has been shown to inhibit the penicillin-binding protein 2a in MRSA strains (Santiago et al., 2015).

Regulation of bacterial gene expression has been proposed as an alternative mechanism of action by phytochemicals, either through modulation of transcription factors or direct interaction with DNA. The antibacterial effect of Caffeic Acid Phenethyl Ester (CAPE) against *E. faecalis*, *L. monocytogenes* and *S. aureus* has been also related to its ability to target RNA- and DNA-related molecules (Murtaza et al., 2014). Naringenin was able to bind the DNA of *S. aureus* ATCC 6538, resulting in major metabolic changes (Wang et al., 2017). Furthermore, the effect of phytochemicals on biofilm formation has been reported: for example, we have shown a dose-dependent effect of phloretin on biofilm production of *S. aureus* (Mandalari et al., 2023) and a dose-dependent effect of a white grape juice extract on biofilms formation of *E. coli* and *Pseudomonas aeruginosa* (Filocamo et al., 2015). Another antibacterial effect of phytochemicals is related to the alteration of the level of bacterial metabolites, proton and ion equilibrium and adenosine triphosphate (ATP) synthesis inhibition, which could determine cell death (Lin et al., 2005; Engels et al., 2011). Certain polyphenols, such as gallic or caffeic acid, could reduce cytochrome activity and, therefore, oxidative phosphorylation, thus inhibiting bacterial growth (Shetty and Wahlqvist, 2004; Kwon et al., 2007).

It is worth noting that the antibacterial effect of polyphenolic plant extracts is often the result of synergistic, indifferent, or antagonistic interactions among the individual compounds. For example, we have demonstrated that bergamot (*Citrus bergamia*) fractions and the pure phytochemical compounds, neohesperidin, hesperetin (aglycone), neoeriocitrin, eriodictyol (aglycone), naringin and naringenin (aglycone), were active against Gram-negative bacteria (*E. coli, Pseudomonas putida, Salm. enterica*) with MIC values in the range 200 to 800 μg/mL. However, pairwise combinations of eriodictyol, naringenin and hesperetin showed synergistic and indifferent interactions, dependent on the selected, tested organism (Mandalari et al., 2007). Moreover, isolated phytochemicals and extracts are being used in combination with traditional antibiotics to sensitize multidrug-resistant bacterial strains (Hatano et al., 2005; Betoni et al., 2006). We have demonstrated a synergistic and post-antibiotic effect of tobramycin in combination with tea tree (*Melaleuca alternifolia*) oil against *S. aureus* and *E. coli* (D’Arrigo et al., 2010). This strategy could represent a valuable tool to combat AMR more effectively.

## 4 Antiviral effect of pistachios and mechanism of action

**Figure 2:**
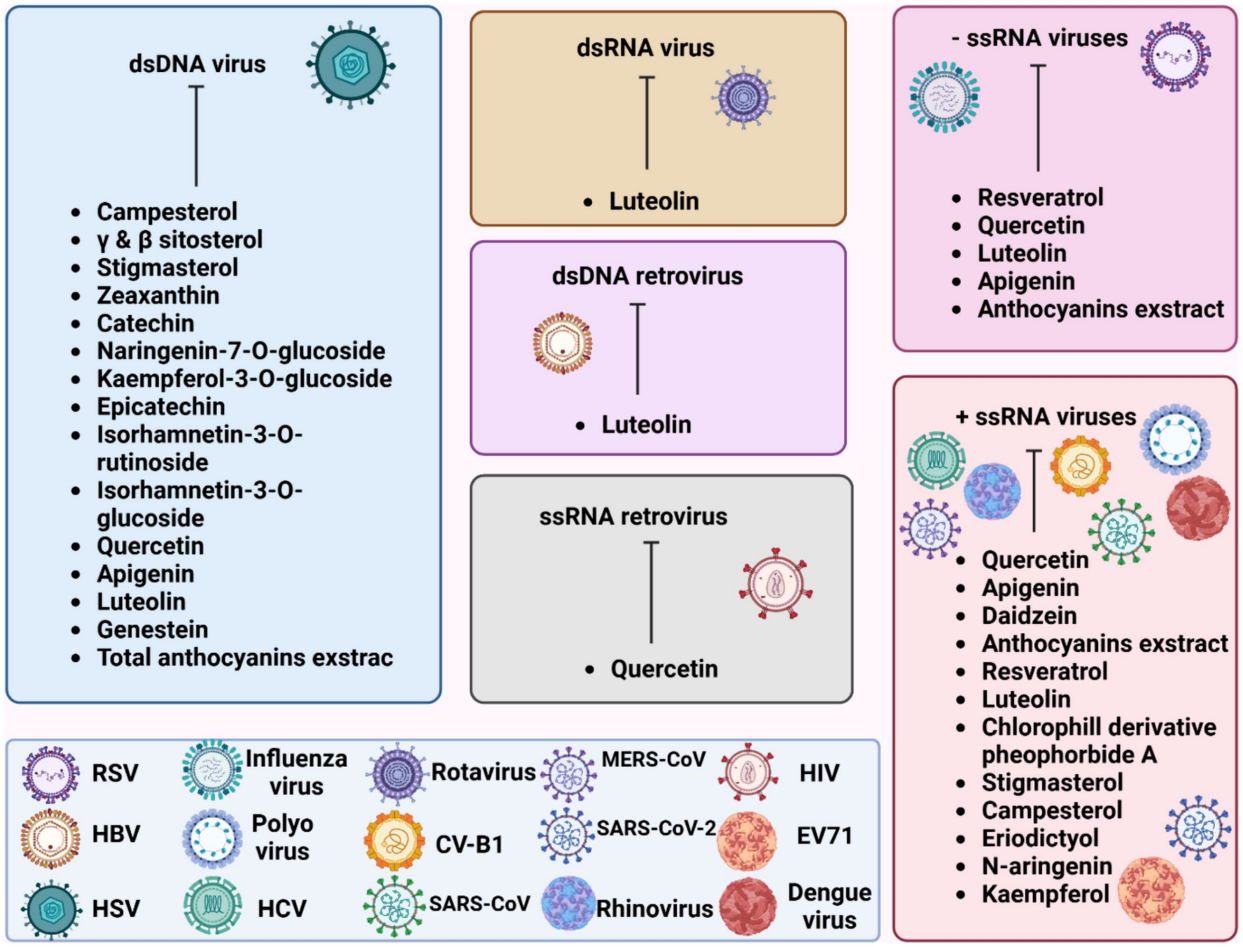
Graphical representation of bioactive natural compounds against viral families.

**Figure 3:**
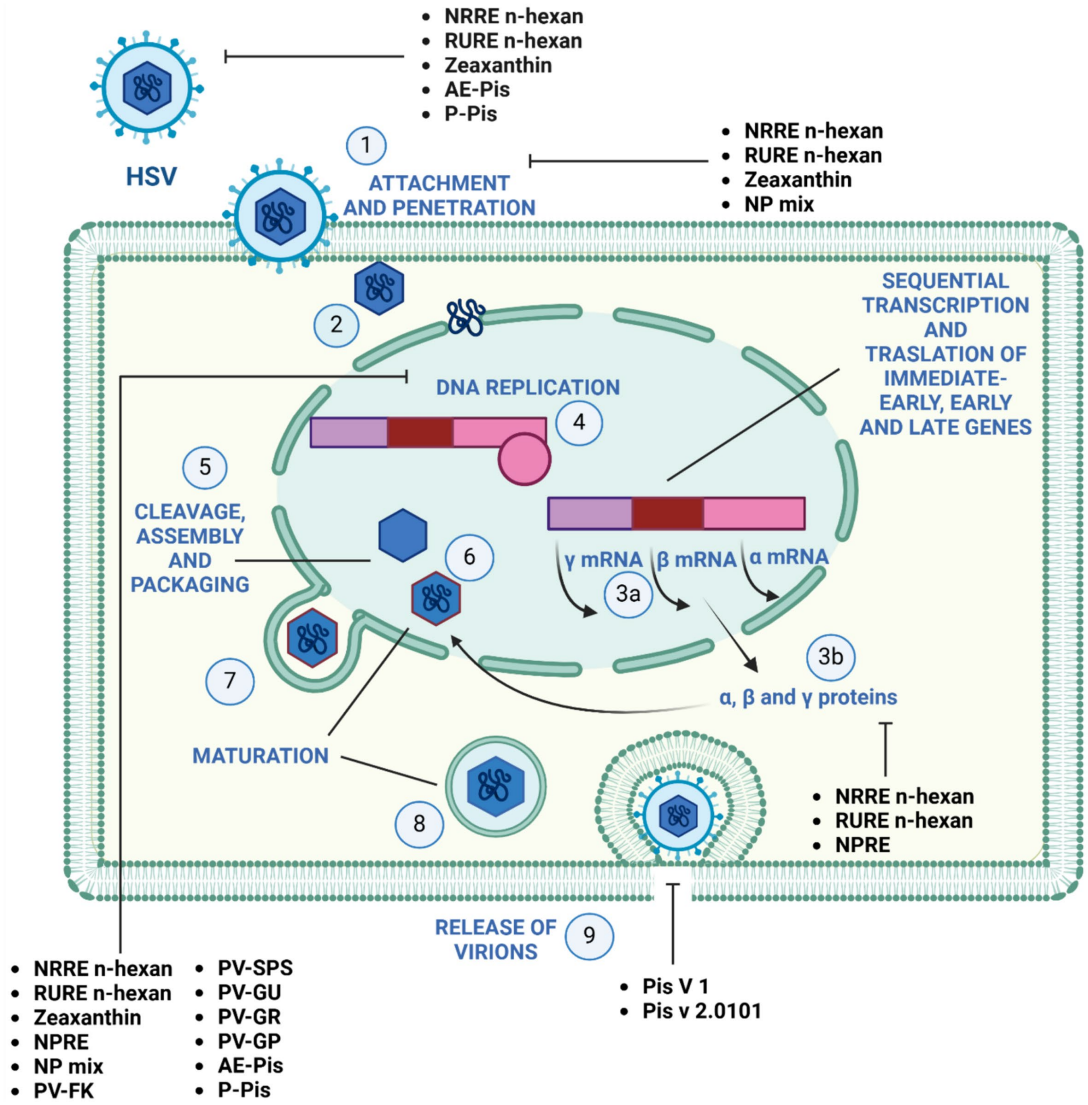
Mechanism of action of pistachio phytochemicals against Herpes simplex virus 1. (1) HSV-1 entry by attachment and penetration. (2) Release of viral DNA. (3) Sequential transcription **(A)** and translation **(B)** of viral immediate-early, early, and late genes. (4) DNA replication by rolling circle mechanism. (5) Assembly and packaging. (6) Maturation and (7) budding of the nucleocapsid out of the nucleus, (8) maturation and (9) release of the virions by exocytosis.

Figure 2 illustrates the effectiveness of bioactive natural compounds against viral families. The antiviral potential of pistachio polyphenolic extracts, particularly against herpes simplex virus type 1 (HSV-1), has been demonstrated (Table 4). We have previously shown that natural raw pistachio extracts (NRRE) significantly reduced the expression of critical viral proteins, including ICP8 (infected cell polypeptide 8), UL42 (DNA polymerase processivity factor), and US11. This reduction was associated with a decrease in viral DNA synthesis, highlighting the extract’s inhibitory effects on HSV-1 replication (Musarra-Pizzo et al., 2020). More recently, we have further investigated the mechanisms involved in the anti-HSV-1 effect exerted by pistachio extracts (Figure 3): NRRE and roasted unsalted (RURE) pistachio polyphenols-rich extracts blocked virus binding on the cell surface, impaired viral DNA synthesis, and prevented the accumulation of viral proteins (Pennisi et al., 2023). Indeed, by testing six compounds present in pistachio polyphenolic extracts (α, β, and δ tocopherol, β-carotene, luteolin, and zeaxanthin), we have demonstrated that zeaxanthin inhibited HSV-1 replication by affecting both viral internalization and replication. Furthermore, zeaxanthin directly interacted with HSV-1 viral particles, leading to a significant reduction in HSV-1 replication (CC50 16.1, EC50 4.08 μM, SI 3.96). Additionally, Özçelik et al. (2005) explored the antiviral properties of 15 lyophilic extracts from various parts of *Pistacia vera*, including leaves, branches, stems, kernels, shell skins, and seeds. Their research identified kernel and seed extracts as having the most potent antiviral effects against herpes simplex and parainfluenza viruses. Specifically, the fresh kernel (PV-FK) and skin of processed-woody shell (PV-SPS) extracts demonstrated significant activity against DNA viruses, which well compared with acyclovir (Figure 3). The Gaziantep sample-unripe (PV-GU) extract also showed notable antiviral activity. On the other hand, some extracts displayed outstanding activity against RNA viruses, such as PV-GR (Gaziantep sample-ripe) and PV-GP (Gaziantep sample-processed). Chhoud et al. (2022) reported the anti-HSV-2 activity of extracts from *Pistacia vera* male floral buds: the aqueous and polysaccharide extracts from male flower buds exhibited a selectivity index (SI) of 29.12 and 20.25, respectively. The extracts showed virucidal activity against HSV-2, likely by altering the viral membrane or interacting with viral ligands, thus inhibiting their binding to receptors on target cells. Finally, pistachio extracts have shown activity against Coxsackie viruses (CV) B2, B3, B4, and B5. Specifically, two pistachio allergens, 2S albumin (Pis v 1) and 11S globulin (Pis v 2.0101), were found to inhibit these viruses (Taghizadeh et al., 2020).

**Table 4:** Antiviral effect of pistachios extracts.

Compound	Class	Virus	Antiviral effect	SI	Reference
NRRE *n-hexane*	Polyphenolic extracts	HSV-1	Viral inactivation Binding inhibition Viral DNA reduction Viral proteins reduction	9.2	Pennisi et al. (2023)
RURE *n-hexane*	Polyphenolic extracts		Viral inactivation Binding inhibition Viral DNA reduction Viral proteins reduction	4.98	
Zeaxanthin	Carotenoids		Viral inactivation Binding inhibition Viral DNA reduction	3.96	
NPRE	Polyphenol-rich extracts	HSV-1	Viral DNA reduction Viral proteins reduction	3	Musarra-Pizzo et al. (2020)
NP mix	Catechin, eriodictyol-7- O-glucoside, gallic acid, protocatechuic acid, caffeic acid, rutin, and isoquercetin		Binding inhibition Viral DNA reduction	-	
PV-FK	Lipophylic extracts	HSV-1	Viral replication	-	Özçelik et al. (2005)
PV-SPS	Lipophylic extracts	HSV-1		-	
PV-GU	Lipophylic extracts	HSV-1		-	
PV-GR	Lipophylic extracts	HSV-1, Parainfluenza viruses		-	
PV-GP	Lipophylic extracts	HSV-1 Parainfluenza viruses		-	
AE-Pis	Aqueous extracts	HSV-2	Viral replication Viral inactivation	29.12	Chhoud et al. (2022)
P-Pis	Polysaccharide extracts			20.25	
Pis v 1	Allergenic protein Estracts	CV- B2, CV- B3, CV-B4, and CV-B5	Inhibitory activity of virus-induced cytopathogenicity	7.33 (CV- B2) 11.80 (CV-B3) 16.35 (CV-B4) 14.21 (CV-B5)	Taghizadeh et al. (2020)
Pis v 2.0101				17.30 (CV- B2) 18.25 (CV-B3) 23.52 (CV-B4) 20.41 (CV-B5)	

NRRE *n*-hexane: *n*-hexane-extracted Californian natural raw pistachio polyphenols-rich extracts; RURE *n*-hexane: *n*-hexane-extracted Californian roasted raw pistachio polyphenols-rich extracts; NPRE: natural shelled pistachios kernels; NP mix: natural shelled pistachio kernels mix; PV-FK: *Pistacia vera* fresh kernel; PV-SPS: *Pistacia vera* skin of processed-woody shell; PV-GU: *Pistacia vera* Gaziantep sample-unripe; PV-GR: *Pistacia vera* Gaziantep sample-ripe; PV-GP: *Pistacia vera* Gaziantep sample-processed; AE-Pis: Aqueous extract from *Pistacia vera* L. male floral buds; P-Pis: Polysaccharide from *Pistacia vera* L. male floral buds; HSV-1: Herpes Simplex Virus type 1; HSV-2: Herpes Simplex Virus type 2; CV: Coxsackie viruses; SI: selectivity index: the ratio of EC50/CC50.

## 5 Conclusion

Due to the increased rates of resistance to antibiotics and antivirals, scientific research is continuously developing to find novel cost-effective alternatives to reduce hospitalization and mortality rates. Amongst natural compounds, the phytochemicals present in pistachio nuts have been shown to exhibit significant antibacterial and antiviral activity against resistant and non-resistant strains. Some findings suggest that the antimicrobial and antiviral effects of pistachio polyphenolic extracts are the result of a balance of the individual bioactive compounds which in combination exert the activity. The synergistic interaction of certain phytochemicals with selected antibiotics or antiviral drugs could be a useful tool to overcome resistance. Nevertheless, isolated compounds, such as zeaxanthin, exhibit strong antiviral activity against HSV-1, affecting viral attachment, penetration and viral DNA synthesis.

While the mechanism of action of pistachio extracts has been partly elucidated, further studies are required to identify more bioactive compounds responsible for the observed effect. However, based on the existing evidence, the use of pistachio extracts and derivatives should be encouraged for the topical treatment of *S. aureus* skin infections and ocular herpetic infections. Existing challenges in the development of antimicrobials from natural products, including cytotoxicity, production of highly active standardized extracts with defined mechanism of action under GMP conditions, and identification of bioactive components taking into account possible interaction amongst the individual compounds, should also be considered, together potential sustainability issues related to pistachio cultivation.

## Author contributions

GM: Conceptualization, Data curation, Funding acquisition, Project administration, Writing – original draft, Writing – review & editing. RP: Writing – review & editing. TG: Writing – review & editing. MS: Writing – review & editing.

## Footnotes

^1^U.S. Department of Agriculture, Agricultural Research Service. FoodData Central. Available online: fdc.nal.usda.gov (Accessed December 16, 2023).

^2^Nutrient Data Laboratory; Beltsville Human Nutrition Research Center Agricultural Research Service. USDA Database for the Flavonoid Content of Selected Foods Release 3.3. Available at: https://www.ars.usda.gov/ARSUserFiles/80400535/Data/Flav/Flav3.3.pdf (Accessed December 16, 2023).

^3^Nutrient Data Laboratory; Beltsville Human Nutrition Research Center; Agricultural Research Service; USDA Database for the Proanthocyanidin Content of Selected Foods Release 2.12018 Available at: https://www.ars.usda.gov/ARSUserFiles/80400535/Data/PA/PA02-1.pdf (Accessed December 16, 2023).

^4^US Department of Agriculture USDA, http://www.ars.usda.gov/nutrientdata.

^5^Phenol-Explorer database http://phenol-explorer.eu/contents.
